# The environmental microbial retrieving assessment of cell-processing facilities for cell therapy in a hospital laboratory

**DOI:** 10.1128/spectrum.01257-24

**Published:** 2024-08-21

**Authors:** Jiabian Lian, Xiaobo Ma, Xun Li, Lu Xia

**Affiliations:** 1 Xiamen Cell Therapy Research Center, The First Affiliated Hospital of Xiamen University, School of Medicine, Xiamen University, Xiamen, China; 2 Center for Precision Medicine, The First Affiliated Hospital of Xiamen University, School of Medicine, Xiamen University, Xiamen, China; 3 Department of Laboratory Medicine, The First Affiliated Hospital of Xiamen University, School of Medicine, Xiamen University, Xiamen, China; Duke University, Durham, North Carolina, USA

**Keywords:** cell therapy clean room, environmental microbiology, metagenomic sequencing, preventive medicine, environmental monitoring

## Abstract

Cell therapy represents a promising treatment modality. A critical component in the production of cell therapy products is maintaining the sterility of cell therapy clean rooms (CTCRs). This study aimed to evaluate the environmental microbial load within CTCRs. We systematically monitored microbial load in CTCRs, following established guidelines. Cultured microbial samples underwent metagenomic sequencing, and alpha and beta diversity analyses, functional annotation, and resistance gene profiling were performed using various bioinformatics tools to assess microbial diversity and function. From November 2023 to January 2024, we collected 42 environmental microbial colony samples from various sources within the CTCR and performed metagenomic sequencing on 39 samples. Alpha diversity analysis revealed no significant differences among surface, settle_plate, and airborne categories, but significant disparities within surface subgroups were revealed. Beta diversity analysis showed notable differences between surface and airborne categories and among surface subgroups. Species distribution analysis identified *Bacillus* as the predominant genus on surfaces. Functional annotation and resistance gene analysis indicated distinct resistance patterns, with significant variations between subgroups, such as microscopes and transfer windows, and hands and other Grade_B environments. Resistance to hydrogen peroxide was notably higher in the transfer window group. These findings highlight the importance of stringent disinfection protocols and enhanced hand hygiene to maintain sterility in CTCRs. These findings provide valuable insights for implementing effective measures to maintain cleanliness throughout CTCRs. The annotation and study of resistance genes can help rapidly identify methods to control cellular contamination under circumstances of environmental microbial pollution.

**IMPORTANCE:**

Maintaining the sterility of cell therapy clean rooms (CTCRs) is crucial for the production of safe and effective cell therapy products. Our study systematically evaluated the environmental microbial load within CTCRs, revealing significant microbial diversity and distinct resistance patterns to disinfection methods. These findings underscore the need for stringent disinfection protocols and enhanced hand hygiene practices to ensure CTCR sterility. By identifying key microbial species and their resistance genes, our research provides essential insights into controlling contamination and safeguarding the production environment, ultimately contributing to the reliability and success of cell therapy treatments.

## INTRODUCTION

The safety of cellular products is compromised not only by undesirable cell characteristics like tumorigenicity but also by contamination from microorganisms during cell culture. Sterilization, while effective, is not feasible for living cell products. Consequently, ensuring the sterility of the cell processing procedure is paramount for safety. The sterility of cell products is ensured through appropriate management of cell therapy clean room (CTCR) environments and traceability based on these records. Environmental management policies should be tailored to the CTCR structure to maintain cleanliness and prevent extrinsic contamination of the process. The risk of such contamination is influenced by the CTCR’s location, both its internal and external environments, and the level of human interaction during the process (1). Key environmental risk factors include temperature, humidity, particle count, entry frequency into clean rooms, airborne microorganisms, and the presence of harmful insects. Among these factors, microbial contamination poses the most direct risk.

The pharmaceutical industry faces stringent requirements for environmental monitoring, particularly in cell therapy and other biotechnological pharmaceutical production processes, where microbial contamination can compromise product purity and safety (2). Mizuno *et al.* usd polymerase chain reaction (PCR) analyses to identify the species of microbial contamination (3). Recent advancements have shifted toward employing culture-independent high-throughput sequencing methods to provide a comprehensive overview of microbial communities within manufacturing facilities, crucial for maintaining stringent quality controls (4, 5). This methodological shift enhances our understanding of microbial dynamics and their implications on production processes. By integrating metagenomic data, manufacturers can better understand and control these microbial communities to prevent product contamination. The continuous evolution of microbial populations necessitates ongoing adjustments to monitoring strategies to maintain compliance with regulatory standards and ensure the production of safe biopharmaceutical products.

The China National Medical Products Administration requires the identification of microbial contamination down to the species level during the production of cell therapy products. The commonly used matrix-assisted laser desorption/ ionization time of flight mass spectrometry (MALDI-TOF-MS) method necessitates the purification and isolation of single colonies. Our research offers a solution for accurate tracing of environmental microbes using metagenomic sequencing. This approach eliminates the need for pure culture isolation, allowing the simultaneous identification of all colonies on a culture plate. In short, we have combined classic culture-based methods with metagenomic sequencing to perform environmental quality control in a CTCR. This integrated approach aims to precisely trace environmental microorganisms.

## MATERIALS AND METHODS

### Classification of areas in the cell therapy clean rooms (CTCR)

**Fig 1:**
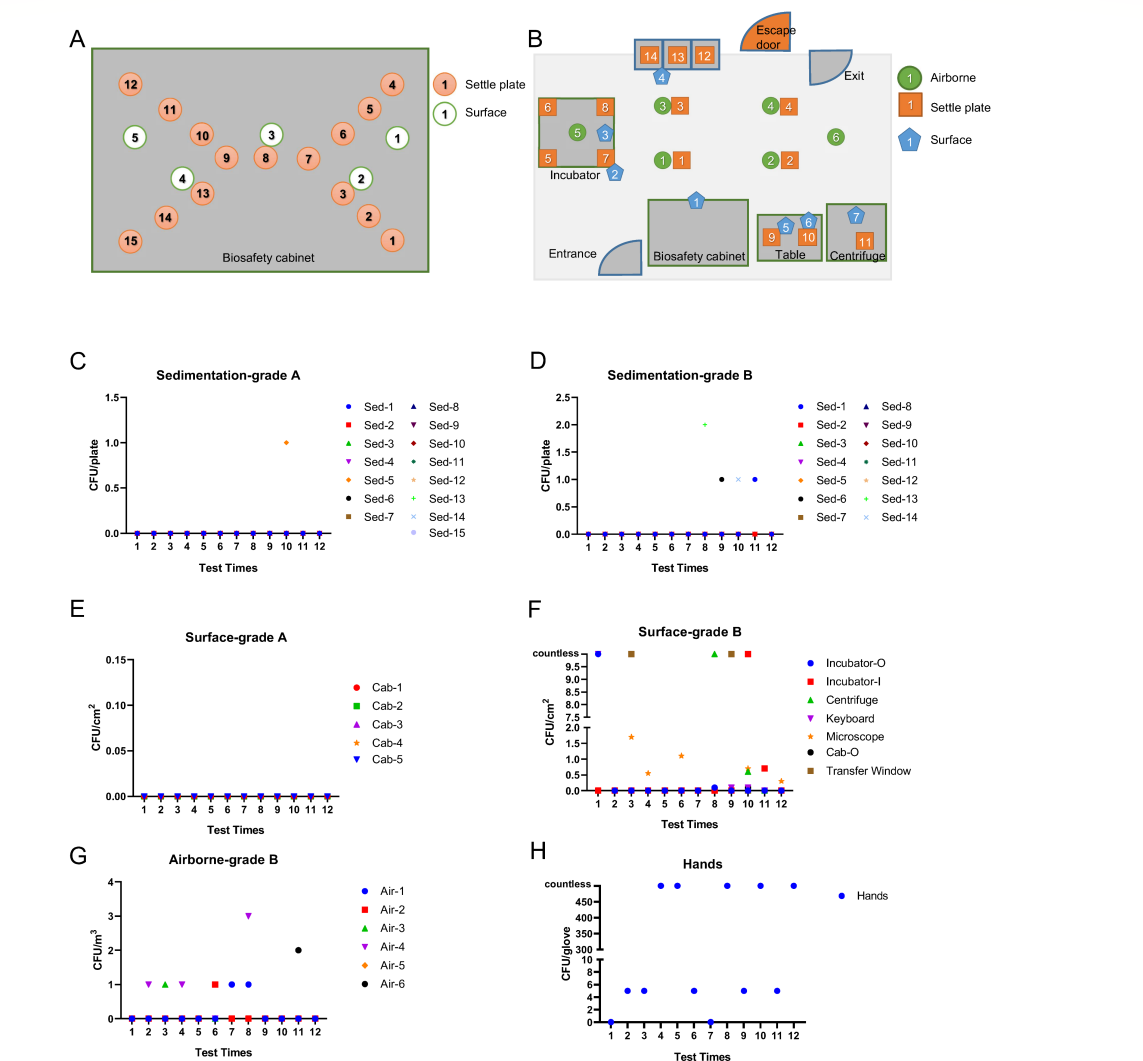
Distribution of sampling points and scatter plots of 12 samplings at different locations. (**A**) Schematic of sampling points in the core area of the cleanroom (Grade_A + Grade_B). (**B**) Schematic of sampling points inside the biosafety cabinet (Grade_A). (**C**) Scatter plot of settling bacteria inside the biosafety cabinet. (**D**) Scatter plot of settling bacteria in Grade B. (**E**) Scatter plot of surface microbes inside the biosafety cabinet. (**F**) Scatter plot of surface microbes in Grade B. (**G**) Scatter plot of airborne bacteria in Grade B. (**H**) Microbial scatter plot on the surface of the operator’s hands. (**C–H**) The x-axis represents the test numbers 1–12 corresponding to dates: 20231109, 20231110, 20231120, 20231122, 20231124, 20231127, 20231129, 20231201, 20231213, 20231227, 20240110, and 20240117.

Figure 1 shows the schematic diagram of our CTCR. The space in the safety cabinet was labeled Grade A, and the other space in the CTCR was labeled Grade B.

### Monitoring of microorganisms

The sampling point layout was conducted in accordance with GB/T 16294–2010, “Test Methods for Settling Microorganisms in Clean Rooms and Associated Controlled Environments of the Pharmaceutical Industry.”; GB 15982–2012, “Hygiene Standards for Disinfection in Hospitals”; WS/T 313–2019, “Standards for Hand Hygiene for Healthcare Workers”; Standard Operation Procedures for Drug Inspection in China, 2019 Edition—Sterility Testing; Pharmacopoeia 9205 (2020), “Guidelines for Microbial Monitoring and Control in Pharmaceutical Clean Laboratories.” All sampling points mentioned in the present study were sampled on the cell production day.

#### Airborne microorganisms’ collection method

Passive sampling, which involves natural sedimentation by gravity, was employed. In the Grade A environment (within biosafety cabinets), 15 sampling points were established with sampling every time before use (Fig. 1A, orange dots), while sampling was conducted at three points during each operation of cell culture (Fig. 1A, green circle 1, 3, and 5). In the Grade B environment, 14 dynamic sampling points were set during each operation (Fig. 1B, orange square). Additionally, for airborne microorganisms in Grade B environments, 1,000 liters of air was aspirated using an airborne microorganism sampler (Suzhou Jingxin, SX-JCQ-5) at six sampling points (Fig. 1B, green dots), with one collection per point during each operation.

#### Hand swab collection method

Fingers were held together and swabbed from the base to the fingertips and back twice using a sterile swab moistened with sterile elution fluid, covering an approximate area of 30 cm² per hand, while simultaneously rotating the swab.

#### Surface sampling method for other objects

For surfaces smaller than 100 cm², the entire surface was swabbed; for surfaces larger than or equal to 100 cm², a 100 cm² area was sampled (Fig. 1A, green circle; Fig. 1B, blue pentagon). A sterile 5 cm × 5 cm template was placed on the object’s surface, and a cotton swab moistened with sterile 0.03 mol/L phosphate-buffered saline (PBS) or physiological saline was used to swab the area within the template vertically and horizontally five times each, followed by rotation of the swab. This process was repeated for four template areas. The part of the swab handled by the operator was cut off, and the swab was placed into a tube containing 10 mL of the sampling fluid for analysis. For irregularly shaped or small objects like door handles, or surfaces smaller than 100 cm², the entire surface was swabbed with a moistened swab head without using a template. All sampling locations are indicated in Fig. S6 to S13.

#### Elution and inoculation

The tip of the swab was then broken off into a “composite neutralizing elution fluid” (Chongqing Pangtong Medical Instruments Co., Ltd., China). The tube containing the swab tip was tightly sealed and vigorously shaken on a vortex mixer for at least 30 seconds to elute the sample, which was then immediately inoculated. A 1-mL aliquot of the neutralizing elution fluid was evenly spread on Columbia blood agar medium by using an inoculation loop. The inoculated agar was placed upright in an incubator for 1 hour to be absorbed and then inverted for further incubation.

From 9 November 2023 to 17 January 2024, we collected a total of 42 environmental microbial colony samples from diverse sources in the CTCR shown in Fig. 1. All samples were collected from one CTCR. Microorganisms were cultured on Columbia blood agar medium at 35°C under aerobic conditions (Chongqing Pangtong Medical Instruments Co., Ltd.). Colony quantification was visually evaluated after 5 days, and images of the colonies were captured using a Huawei P20 cellphone.

### Identification of bacterial and fungus species by metagenomic sequencing

#### Extraction of microbiome DNA

Colonies of microorganisms were manually picked up. The sample, ranging from 100 to 200 mg, is transferred to a centrifuge tube containing grinding beads and 1 mL of buffer ATL/PVP-10 (MagPure Stool DNA Kit #D636402, Magen, China). After grinding, the sample was incubated at 65°C for 20 minutes, centrifuged, and the supernatant is mixed thoroughly with 0.6 mL of buffer PCI (Magen, China). Following another centrifugation, the DNA, now in the supernatant, is transferred to a deep well plate prepared with magnetic beads (Magen, China). The procedure for microbiome DNA extraction begins with the preparation of five 96-well deep plates containing 600 µL of a buffer with magnetic beads, 20 µL of proteinase K (Magen, China), and 5 µL of RNase A (Magen, China) for each well, followed by three wash steps each with 700 µL of Wash 1, Wash 2, and Wash 3, and ending with the addition of 100 µL of elution buffer (Magen, China). The plate is then placed in a Kingfisher machine (Thermo Fisher, USA) to bind and wash the DNA, which is finally eluted into a 1.5-mL centrifuge tube.

#### Metagenome library preparation and sequencing

The metagenome library preparation uses the MGIEasy Universal DNA Library Prep Set (MGI, China). The genomic DNA is then randomly fragmented, and fragments of a specific average size are selected. The fragment processing includes end repair, adenine addition, and adapter ligation. A PCR amplification step follows to enrich the DNA library, which undergoes a final QC to confirm its suitability for sequencing. The library is then subjected to circularization, forming single-stranded circle DNA that is used to create DNA nanoballs (DNBs) via rolling cycle amplification. These DNBs are loaded onto patterned nanoarrays and sequenced using combinatorial Probe-Anchor Synthesis (cPAS) technology (MGI, China). DNA quantification is conducted using the Qubit dsDNA BR Assay Kit or Qubit ssDNA Assay Kit (Thermo Fisher Scientific, USA). MGISEQ-2000 (MGI, China) was applied for sequencing.

#### Metagenomic data analysis

The initial stage of metagenomic data analysis involves the filtering of raw sequencing data using SOAPnuke (v2.2.1) (6) to remove reads with adapter sequences, high N base content, and low-quality bases. The clean data are assembled *de novo* using MEGAHIT (7). Gene prediction is performed on the assembled sequences using MetaGeneMark (8), followed by clustering with CD-HIT to reduce redundancy (9). Gene abundance is quantified using Salmon to generate normalized TPM values (10). To annotate protein sequences, they were matched against various functional databases such as BacMet (Antibacterial Biocide and Metal Resistance Genes Database; version：20180311), CARD (The Comprehensive Antibiotic Resistance Database; version：3.0.9), and KEGG (Kyoto Encyclopedia of Genes and Genomes; version：101), utilizing the DIAMOND tool (11). Taxonomic annotation was performed using the Nt (202011) database and the Kraken (12) LCA algorithm. For creating taxonomic and functional abundance profiles, the Bracken software (https://github.com/jenniferlu717/Bracken) was employed with its default settings. Features like genera, phyla, and KOs showing significant differential abundances between groups were identified through Wilcoxon’s rank sum test (13). Alpha diversity indices, such as Shannon, Chao1, and Simpson, were calculated at the levels of gene, genus, and KO using their relative abundances in the R package. Beta diversity was assessed through metrics like Euclidean distance or Bray–Curtis distance. Statistical tests, including the Wilcoxon rank test and Kruskal–Wallis H test, were performed using the R project. The results of the intergroup functional differences analysis were determined and visualized using the STAMP software (14). This comprehensive analysis aids in understanding the diversity and function of microbial communities in the sample.

## RESULTS

### Sampling and culturing of environmental microorganisms

We collected a total of 42 environmental microbial colony samples from diverse sources (Fig. 1), including airborne bacteria (*N =* 8), settled bacteria (*N =* 4 for Grade_B and *N =* 1 for Grade_A), the operators’ hands (*N =* 11), and various surfaces during the operational process such as the handlers of the biosafety cabinet (Cab_O, *N =* 1), the inside of the incubator (Incubator_I, *N =* 2), the incubator door (Incubator_O, *N =* 2), microscope (*N =* 6), transfer windows (*N =* 2), keyboard and mouse (*N =* 3), and centrifuge (*N =* 2). The most frequent detections occurred on the operators'’ hands, airborne bacteria, and microscopes. We extensively collected and sequenced microbial colonies from culture plates, successfully analyzing 39 samples (Table S1). These findings underscore the critical need for rigorous microbial monitoring in ensuring the quality of cell therapy products. By tracing microbial sources and calculating the frequencies of microbial occurrence at various sampling points over time, we can implement targeted interventions to mitigate contamination risks before they spread, thereby enhancing the safety of cell-based therapies.

### Metagenomic sequencing and genetic diversity analysis

**Fig 2:**
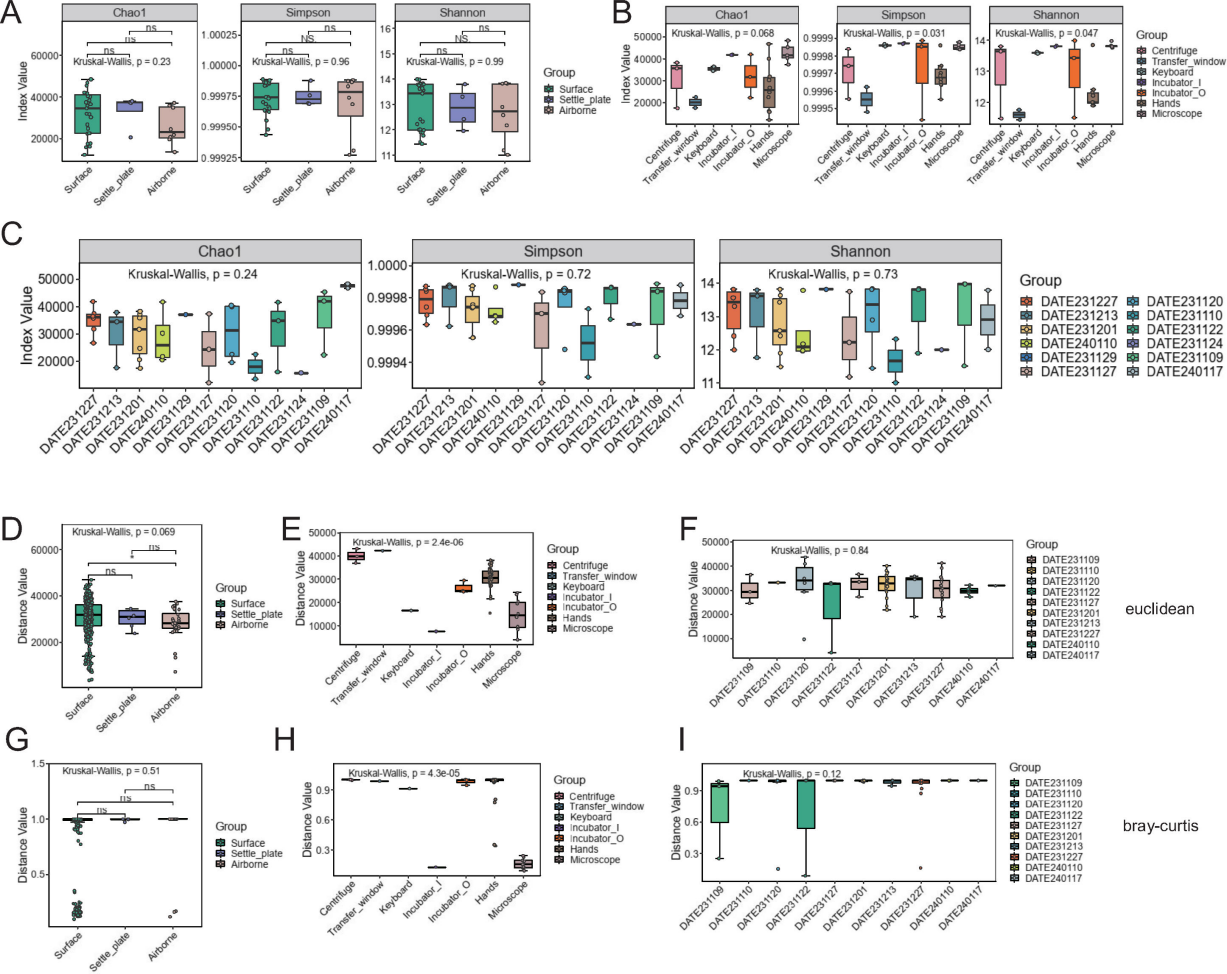
Genetic diversity and variation in environmental microbial samples. (**A–C**) Grouping samples by different sampling locations (**A and B**) and dates (**C**) produces box plots of gene alpha diversity. Each box plot represents a diversity index, with the x-axis and the boxes indicating groups, and the y-axis shows index values. Hypothesis testing methods and results are noted above each plot. (**D–I**) Box plots of gene beta diversity by sampling location (**D and E, G and H**) and date (**F and I**). The x-axis and box colors indicate groups, while the y-axis shows the distance between samples. The upper and lower edges of the boxes represent the upper and lower quartiles of within-group distances, respectively, with the median marked by a horizontal line inside the box. The ends of the lines above and below the boxes indicate the maximum and minimum values, respectively. Each box reflects the distribution of distances within its group, with larger medians suggesting greater within-group sample dispersion. Longer boxes and lines outside the boxes indicate greater within-group variance. The distance matrices use Euclidean distance (**D–F**) or Bray–Curtis dissimilarity (**G–I**) to measure between-group differences. When the number of groups is 3 or 4, a line above the boxes connects two groups, with statistical test indicators or *P*-values above this line: **** for *P <* 0.0001, *** for *P <* 0.001, ** for *P <* 0.01, * for *P <* 0.05, ns for *P >* 0.05, and NS for *P =* 1.

We assessed the genetic diversity, starting with alpha diversity measures (Fig. 2A through C). Alpha diversity, indicative of species richness and evenness within an ecosystem, was quantified using Chao1, Shannon, and Simpson indices. No significant differences were noted among surface, settle_plate, and airborne categories using these indices (grouping information in Table S1). However, a further breakdown of the surface group into seven subgroups revealed significant disparities in Shannon and Simpson indices. Additionally, temporal grouping showed no significant variations in these indices. Beta diversity analysis explored genetic disparities across categories, identifying significant differences between surface and airborne categories and among the subgroups of the surface category (Fig. 2D through I）. Temporal analysis again showed no significant variations. These genetic diversity assessments underscore the importance of understanding microbial ecosystems in cell therapy environments since variations in microbial communities must be documented due to their potential impact on product safety in the event of contamination.

### Species diversity analysis

**Fig 3:**
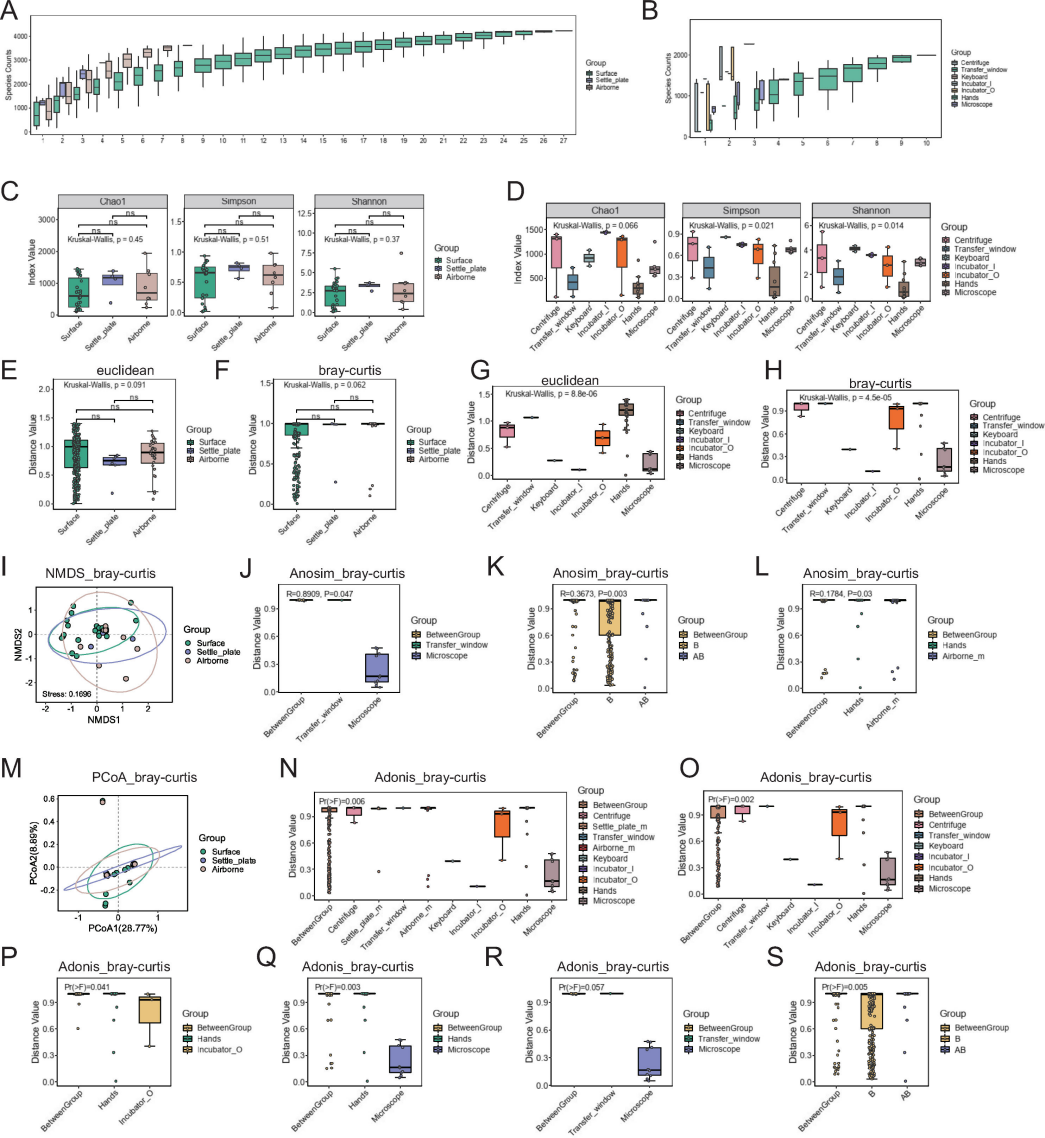
Species alpha and beta diversity of environmental microbial samples at the species level. (**A and B**) Rarefaction curve box plots for different grouping methods. The x-axis indicates the number of samples, and the y-axis indicates the number of species detected, with the colors of the boxes representing groups. Each box shows the species count achievable at a given sample size, with the upper and lower edges representing the upper and lower quartiles of within-group distances, respectively, and a horizontal line inside the box indicating the median. The ends of the lines above and below the boxes indicate the maximum and minimum values, respectively. (**C and D**) Alpha diversity box plots grouped by sampling location, with each box plot representing a diversity index. The x-axis and the boxes represent groups, and the y-axis shows index values. Hypothesis testing methods and results are noted in the upper left corner of each box plot, with *P <* 0.05 indicating significant differences in alpha indices between groups. (**E–H**) Beta diversity box plots grouped by sampling location, with each box plot representing distances between samples. The x-axis and box colors represent groups, the y-axis shows distances, and the box edges represent the upper and lower quartiles of within-group distances. Each box reflects the distribution of distances within its group, with larger medians indicating greater within-group sample dispersion. The distance matrices use Euclidean distance (**E and G**) or Bray–Curtis (**F and H**) to measure between-group differences. When the number of groups is 3 or 4, a line above the boxes connects two groups, with statistical test indicators or *P*-values above this line: **** for *P <* 0.0001, *** for *P <* 0.001, ** for *P <* 0.01, * for *P <* 0.05, and ns for *P >* 0.05. (**I**) NMDS scatter plot based on Bray–Curtis distance. Each point represents a sample, with the color indicating its group. Confidence ellipses (only present for groups with four or more samples) have a 95% confidence level. The quality of NMDS results is defined by stress values, with stress <0.2 indicating interpretive significance. (**J–L**) Anosim analysis based on Bray–Curtis distance, a nonparametric test using permutation and rank sum tests to assess whether between-group differences exceed within-group differences. The y-axis shows sample distances, with “BetweenGroup” indicating between-group distances and the remaining labels indicating within-group distances. Each box describes the distance distribution for different groups, with the upper and lower edges representing the upper and lower quartiles of within-group distances, respectively; a horizontal line inside the box indicating the median; and the ends of the lines above and below the box indicating the maximum and minimum values, respectively. R is the Anosim statistic, with R > 0 indicating higher within-group similarity; R approaching 0 indicating no difference in similarity between and within groups; *R* < 0 indicating greater between-group similarity. The *P*-value, *P <* 0.05, indicates significant between-group differences. (**M**) PCoA scatter plot based on Bray–Curtis distance, with each point representing a sample, the color indicating its group, and the axes showing the highest explanatory dimensions PCoA1 and PCoA2. The axis titles include the percentage of variance explained by these dimensions, and confidence ellipses (only present for groups with four or more samples) have a 95% confidence level. (**N–S**) Adonis analysis based on Bray–Curtis distance, aimed at testing whether there are significant differences between groups. The y-axis shows sample distances, with “BetweenGroup” indicating between-group distances and the remaining labels indicating within-group distances. Each box describes the distance distribution for different groups, with the upper and lower edges representing the upper and lower quartiles of within-group distances, respectively; a horizontal line inside the box indicating the median; and the ends of the lines above and below the box indicating the maximum and minimum values, respectively. *Pr(>F)* is the frequency of permutation tests F greater than the observed F, with *Pr(>F)* <0.05 indicating significant between-group differences.

Species accumulation curves were used to evaluate the adequacy of our sampling efforts and to predict community richness (Fig. 3A and B). Pie charts of dominant species distribution under different grouping conditions and representative environmental microorganisms cultured on CBA plates can be found in Supplementary files (Fig. S1). We assessed alpha diversity across surface, settle_plate, and airborne groups, noting no significant differences; however, distinct variations emerged among the surface subgroups (Fig. 3C and D). Our beta diversity analysis corroborated these findings, showing no temporal differences, but variations existed among the surface subgroups (Fig. 3E through H). Significant intergroup variations were identified using analysis of similarities (Anosim) and permutational multivariate analysis of variance (PERMANOVA, Adonis), revealing notable differences between specific pairs such as microscopes and transfer windows, hands, and other Grade_B environmental groups (Fig. 3I through S). The investigation of species diversity indicates that initial contamination on the surfaces of objects in the newly established CTCR primarily originated from insufficient disinfection of the items themselves.

**Fig 4:**
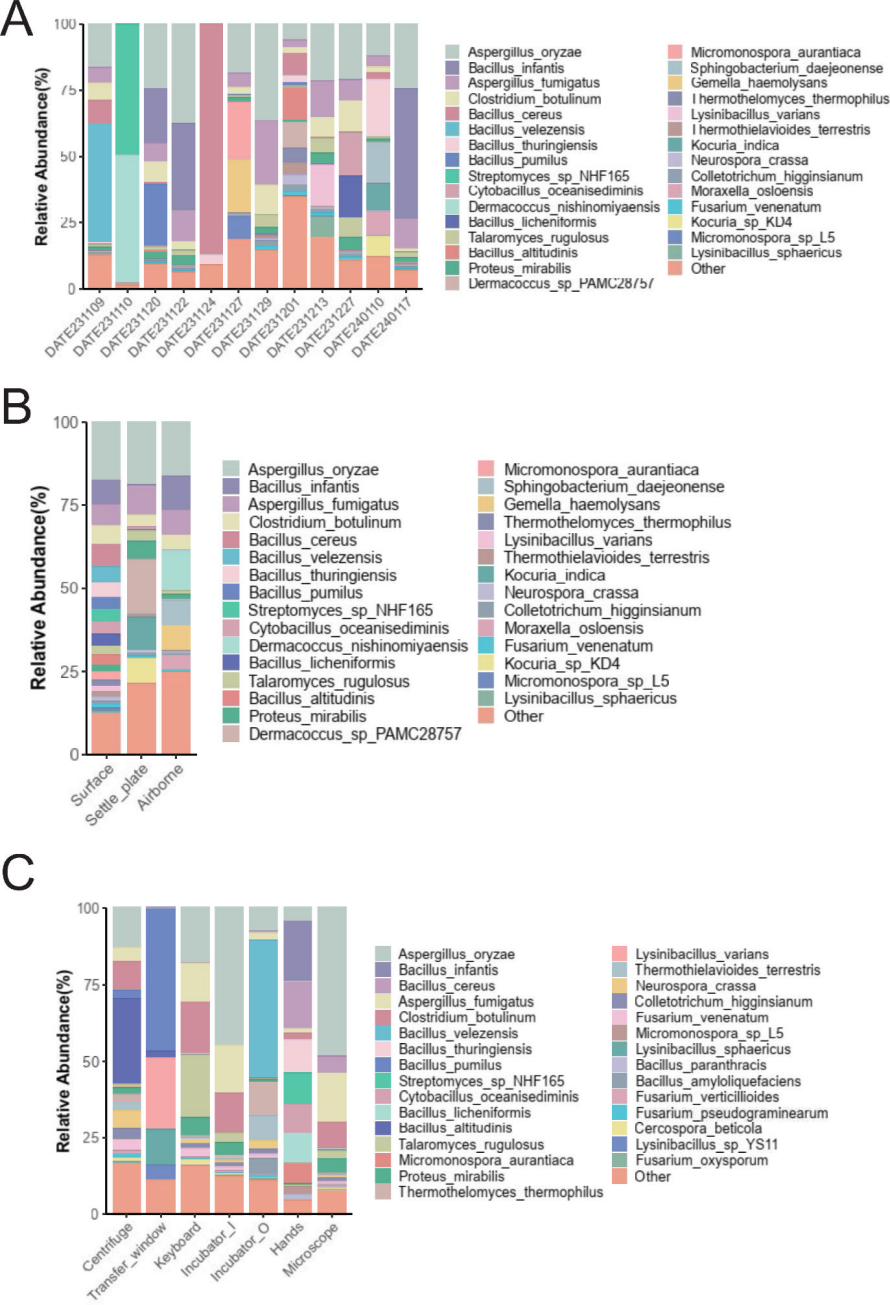
Stacked bar charts of species distribution. (**A**) Grouped by date, (**B**) by sampling method, and (**C**) by surface type. The x-axis shows the sample/group, and the y-axis shows the relative abundance of species, with the colors of the bars representing species classifications. The longer the bar, the higher the relative abundance.

The species distribution within different groups was visually represented through stacked charts and pie charts, detailing the dominant species present in various environments (Fig. 4; Fig. S1). Among them, *Aspergillus*, *Bacillus*, and *Clostridium* are the most common genera of microorganisms. The linear discriminant analysis effect size (LEfSe) tool was employed to pinpoint species that significantly explain differences between two or more groups, serving as biological markers. LEfSe, ideal for multilevel discovery, primarily focuses on species taxonomic phylogeny, plotting the top 300 species in abundance (Fig. S2). The abovementioned analysis consistently indicates that *Bacillus* is the predominant genus distinguishing the surface environments from others. These insights into species diversity are crucial for identifying potential key contamination species and guiding effective control measures in cell therapy manufacturing. In the contamination scenarios described in this study, wiping with a 500 mg/L sodium hypochlorite disinfectant solution gradually eliminated surface contamination issues in subsequent work (Fig. 1F).

### Functional annotation and microbial community resistance

We first performed functional annotation on the metagenome by annotating predicted nonredundant genes to pertinent functional databases and summarizing their abundances. The functional data derived from the Kegg, BacMet, and Card databases are illustrated in stacked distribution charts (Fig. S3 to S5). The annotated KEGG pathways primarily involve various metabolic and biosynthetic pathways, as well as quorum sensing, which may be related to the cultivation process of microbes. The BacMet database annotations identify resistance substances, including arsenic, hydrogen peroxide, and copper. The CARD database annotations indicated antibiotic resistance mainly to penam, cephalosporin, and tetracycline antibiotics. The broad resistance risks suggest that preventing environmental microbial contamination is more effective than using drugs for sterilization after cell contamination has occurred.

**Fig 5:**
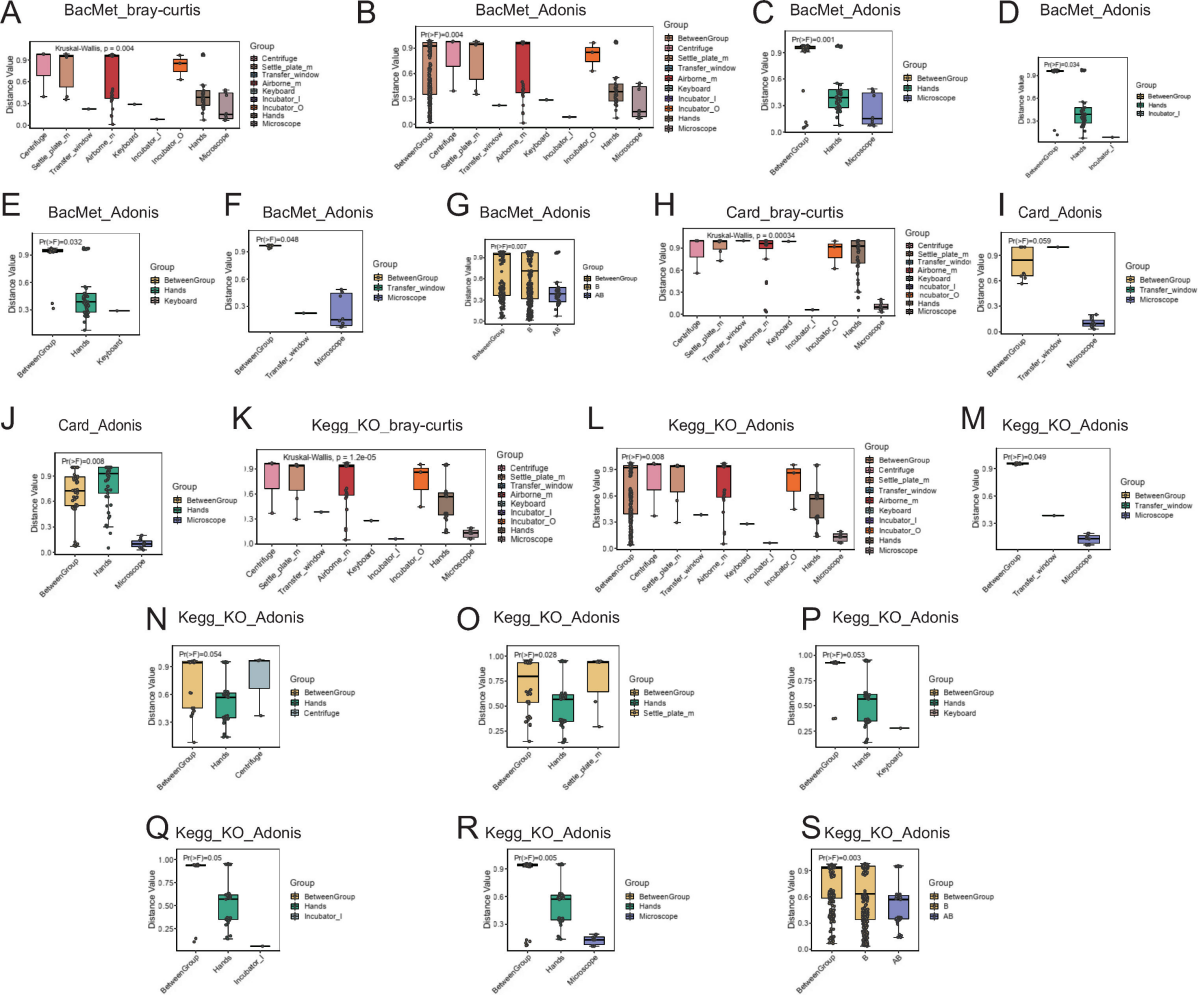
Beta diversity analysis of functions based on Bray–Curtis distance. (**A**) Beta diversity box plot for functions from the BacMet database. (**F and G**) Adonis analysis for functions from the BacMet database. (**H**) Beta diversity box plot for functions from the Card database. (**I and J**) Adonis analysis for functions from the Card database. (**K**) Beta diversity box plot for KEGG database KO-level functions. (**L–S**) Adonis analysis for KEGG database KO-level functions. The x-axis and box colors represent different groups, the y-axis shows the distances between samples, and the box edges represent the upper and lower quartiles of within-group distances. Each box reflects the distribution of distances within its group, with larger medians indicating greater within-group sample dispersion. When the number of groups is 3 or 4, a line above the boxes connects two groups, with statistical test indicators or *P*-values above this line: **** for *P <* 0.0001, *** for *P <* 0.001, ** for *P <* 0.01, * for *P <* 0.05, and ns for *P >* 0.05. The Adonis analysis box plot shows the distances between samples in the y-axis, with “BetweenGroup” indicating between-group distances and the remaining labels indicating within-group distances. Each box describes the distance distribution for different groups, with the upper and lower edges representing the upper and lower quartiles of within-group distances, respectively; a horizontal line inside the box indicating the median; and the ends of the lines above and below the box indicating the maximum and minimum values, respectively. Pr(>F) has the same meaning as the p-value, with Pr(>F) <0.05 indicating significant between-group differences.

**Fig 6:**
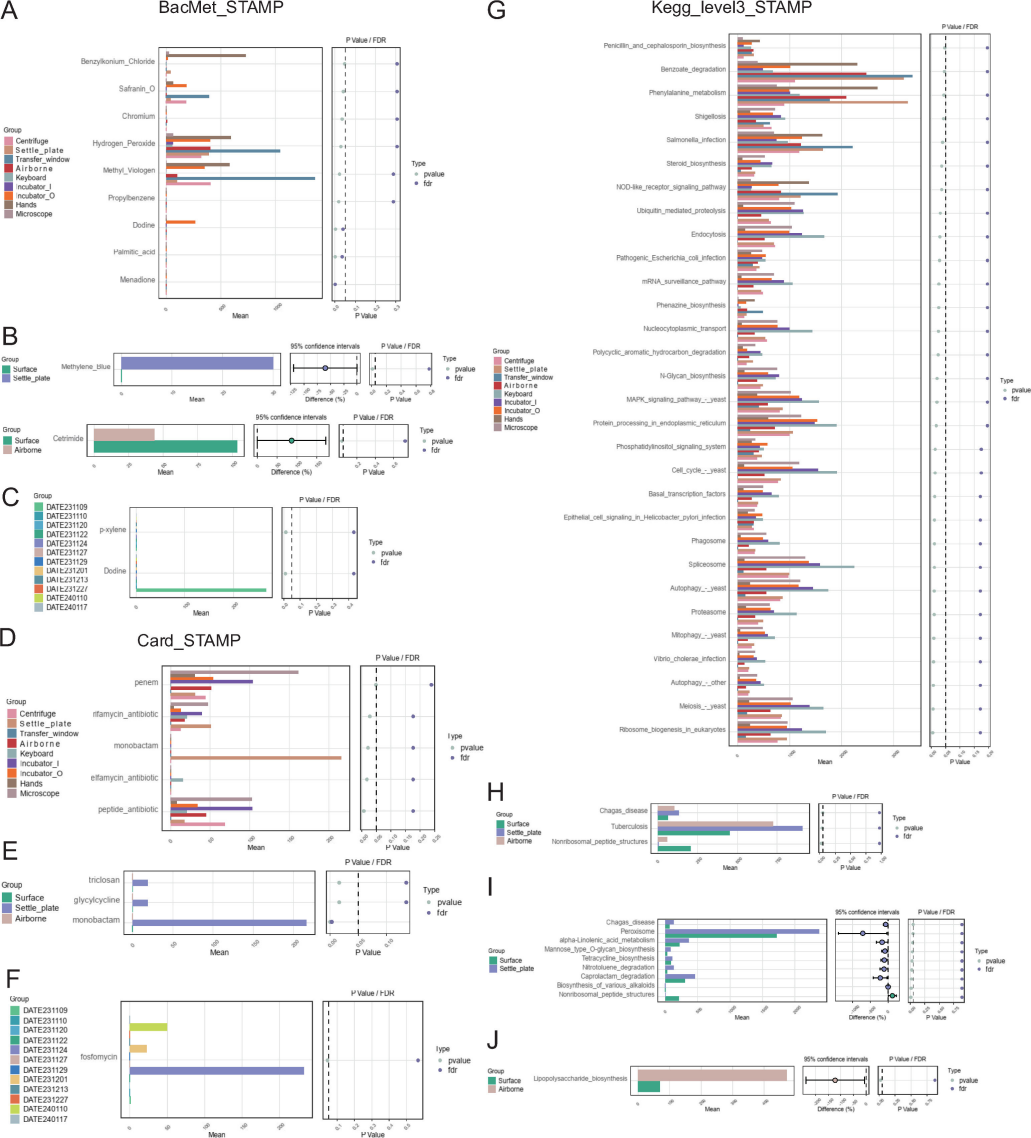
STAMP analysis of functional abundance. (**A–C**) Extended bar charts from the BacMet database. (**D–F**) Extended bar charts from the Card database. (**G–J**) Extended bar charts for level 3 annotations from the KEGG database. STAMP extended bar charts: On the left, the y-axis shows different functions, the x-axis shows group average abundances, and the colors of the bars represent groups; on the right, the scatter plot shows different significance test results, with *P*-value as the statistical test result and FDR as the false discovery rate, which is the adjusted *P*-value. Points to the left of the dashed line (*P <* 0.05) indicate significant differences; the middle area (for two-group comparisons) displays the 95% CI for the difference in abundances between groups, with the position of the dots representing the average difference, and the color of the dots representing the group with higher abundance, connected by lines indicating the confidence bounds. R language is used for nonparametric statistical testing, with results visualized using extended bar charts. If the analysis results include more than 30 different functions, only the top 30 in relative abundance are selected for visualization; if fewer than 30, all are selected.

Subsequently, we carried out beta diversity analysis of functions linked to the BacMet, Card, and KEGG databases (Fig. 5). The Card and BacMet databases catalog resistance genes, their products, and related phenotypes. The samples were categorized into surface, settle_plate, and airborne groups, showing no significant differences among these categories. However, further subdivision of the surface group into seven subgroups revealed notable differences in beta diversity. To assess whether intergroup differences exceeded intragroup variations, we utilized Anosim and PERMANOVA with both Euclidean and Bray–Curtis distance matrices across all groupings (Fig. 5, only Bray–Curtis shown). Significant variations were observed between the microscope and transfer window; between hands and other Grade_B environmental groups with notable intergroup differences in both Euclidean and Bray–Curtis distance matrices. A functional STAMP analysis further explored these intergroup differences, revealing distinct resistance patterns to various antibacterial biocides or metals across different microbial communities (Fig. 6). For instance, Hydrogen_Peroxide resistance was significantly higher in the transfer_window group (Fig. 6A) and monobactam resistance was significantly higher in the Settle_plate group (Fig. 6E), indicating diverse interactions between environmental types inside a cell therapy cleanroom and potential antibacterial agents the operators may apply or avoid. This functional analysis underscores the significance of monitoring microbial resistance patterns to ensure the efficacy of decontamination strategies and maintain the integrity of cell therapy products.

## DISCUSSION

Identifying the diversity of environmental microbial species is crucial for pharmaceutical production that cannot be terminally sterilized. This practice not only helps understand the microbial ecosystems within the production environment but also identifies potential sources of contamination. By conducting detailed analyses of environmental microbes, manufacturers can develop targeted cleaning and disinfection strategies, reducing the risk of contamination and ensuring the safety and efficacy of pharmaceutical products. Additionally, monitoring microbial diversity aids in complying with regulatory requirements and maintaining high standards of production quality. This process enhances production transparency and control, ultimately safeguarding patients’ health.

The use of 16S rRNA sequencing, as employed by Hamdy *et al*., provides valuable insights into microbial communities by targeting a specific region of the bacterial ribosomal RNA gene (5). However, this method has several limitations compared to more comprehensive metagenomic sequencing. First, 16S rRNA sequencing is limited to bacterial identification and does not extend to viruses, fungi, or other microorganisms, which can also be critical contaminants in pharmaceutical environments. This restricts the scope of microbial community analysis to bacteria alone (15). Additionally, 16S rRNA sequencing can introduce biases due to the variability in primer specificity and the presence of multiple copies of the rRNA gene in some bacteria, potentially skewing abundance estimates (16). In contrast, metagenomic sequencing analyzes all of the genetic material present in a sample, providing a more holistic view of the microbial landscape. This method allows for the identification of all microorganisms, including bacteria, viruses, fungi, and protozoa, and offers insights into their functional capabilities, such as antibiotic resistance and metabolic pathways (17). Metagenomic sequencing also avoids the primer bias inherent in 16S rRNA sequencing and does not rely on the amplification of a specific gene region, thus reducing the likelihood of distorted microbial community representations. Therefore, while 16S rRNA sequencing is useful for bacterial surveys, metagenomic sequencing offers a more accurate and comprehensive tool for environmental monitoring in pharmaceutical settings, essential for ensuring the safety and efficacy of biopharmaceutical products.

Metagenomics allows for a broader and more accurate detection of microbial populations, including non-culturable organisms, which are often overlooked yet could pose significant risks (4). However, efforts must be taken to explore a metagenomic DNA sequencing assay that is robust against environmental DNA contamination since metagenomic DNA is sensitive to environmental DNA contamination, in particular when applied to samples with low microbial biomass (18). Both the sequencing and bioinformatics approach used can significantly influence results of a metagenomic DNA sequencing assay (17). To combine the advantage of classic culture methods and metagenomic DNA sequencing assay, our research introduces a significant innovation by incorporating metagenomic sequencing alongside classic methods. “First culture then sequencing” ensure both bacterial and fungi can be identified at the genus level. This novel integration enhances the comprehensiveness and accuracy of microbial assessments in clean rooms dedicated to cellular therapy production. Such an approach is not widely reported in existing literature, positioning our study at the forefront of optimizing environmental quality control. This integration allows for a more detailed understanding of microbial dynamics, essential for maintaining stringent quality controls and ensuring the safety of biopharmaceutical products. Our methodological innovation addresses both the complexity of microbial ecosystems and the economic constraints faced in cellular therapy production, marking a pivotal advancement in environmental monitoring strategies.

While the present “First culture then sequencing” method ensures the detection and identification of microorganisms based on classic sampling and culturing techniques, it cannot detect nonculturable microorganisms. In future studies, researchers could establish a background microbial database for reagents and explore direct sequencing of samples without prior culturing. However, this approach will pose significant challenges to the detection sensitivity of sequencing methods.

### Conclusion

From the microbial source tracking results of this study, several conclusions can be drawn. First, the investigation of beta diversity at different levels indicates that initial contamination on the surfaces of objects in the cell therapy cleanroom primarily originated from insufficient disinfection of the items themselves, rather than from personnel hand manipulation. Subsequently, we effectively controlled bacterial contamination on surfaces such as keyboards, microscopes, and transfer windows using chlorine-based disinfectants. Additionally, since hand contamination was the most frequent occurrence, there is a need to enhance hand hygiene among personnel. This includes strategically placing pedal-operated or sensor-activated running water sinks in the changing rooms and emphasizing the use of alcohol for disinfecting gloves during operations. Furthermore, hand sampling should be conducted only after the disinfecting alcohol has been fully effective and evaporated to prevent its dilution by the components in the neutralizing buffer, which could render the disinfection ineffective. Moreover, the annotation and study of resistance genes can help us rapidly identify methods to control cellular contamination under circumstances of environmental microbial pollution.

## Data Availability

Study data are available upon request. The datasets generated during and/or analyzed during the current study are available in the China National GeneBank DataBase (CNGBdb, https://db.cngb.org/) repository with accession number CNP0005860.

## SUPPLEMENTAL MATERIAL

Supplemental material spectrum.01257-24-s0001.pdf Fig. S1 to S13.

Table S1 spectrum.01257-24-s0002.xlsx Categories of metagenomic sequencing data.

ASM does not own the copyrights to Supplemental Material that may be linked to, or accessed through, an article. The authors have granted ASM a non-exclusive, world-wide license to publish the Supplemental Material files. Please contact the corresponding author directly for reuse.
