# Supplementary material for: The environmental microbial retrieving assessment of cell-processing facilities for cell therapy in a hospital laboratory
Source: Microbiol Spectr. 2024 Aug 21;12(10):e01257-24. doi: 10.1128/spectrum.01257-24 (PMC11448444; doi:10.1128/spectrum.01257-24)
Supplement: Supplemental material — Fig. S1 to S13. [file spectrum.01257-24-s0001.pdf]

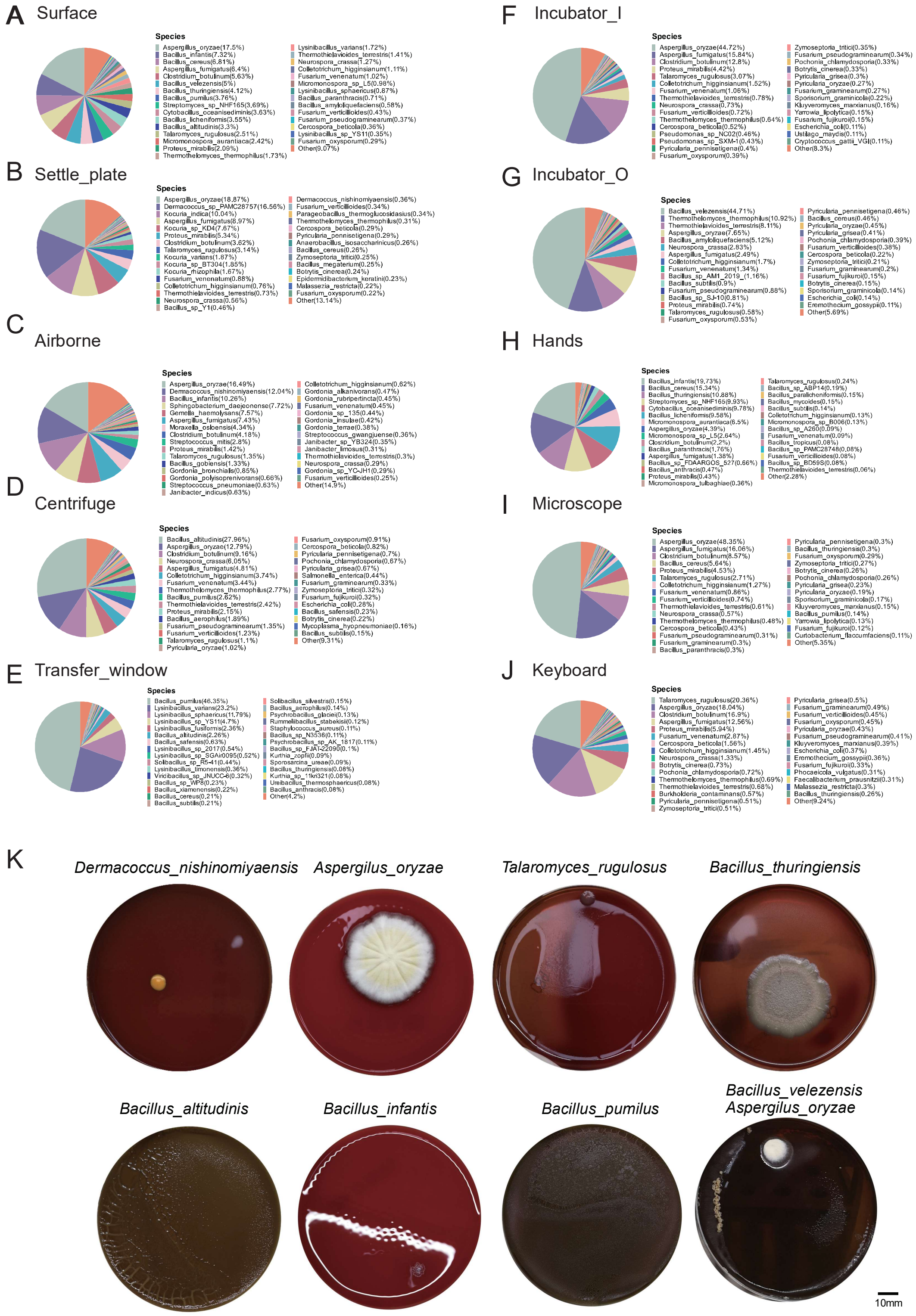
A Surface
B Settle_plate
C Airborne
D Centrifuge
E Transfer_window
F Incubator_I
G Incubator_O
H Hands
I Microscope
J Keyboard
K
Dermacoccus_nishinomiyaensis
Aspergilus_oryzae
Talaromyces_rugulosus
Bacillus_thuringiensis
Bacillus_altitudinis
Bacillus_infantis
Bacillus_pumilus
Bacillus_velezensis
Aspergilus_oryzae
10mm

Supplementary Figure 1. Pie charts of species and representative CBA plate culture morphologies of environmental bacteria. (A-J) Pie charts for different groups, with different sectors and colors representing different species classifications, and the central angles of the sectors representing the proportion of each species in the total. The names of the corresponding groups are marked above the pie charts. (K) Representative CBA plate culture morphologies of environmental bacteria, with species names marked above the chart.

A

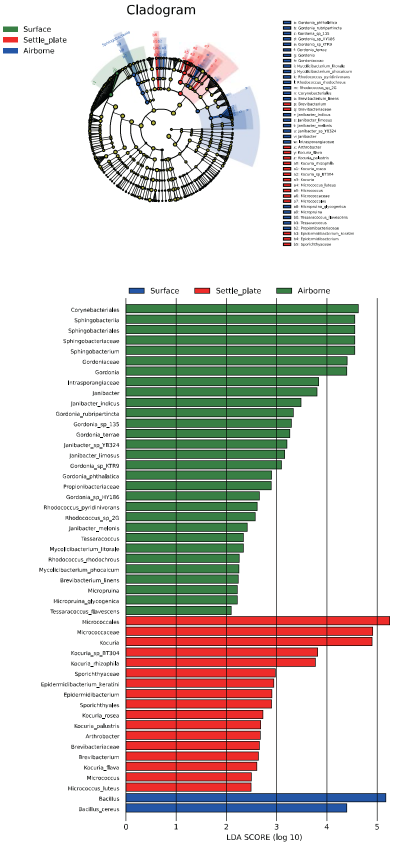
Cladogram
Surface
Settle_plate
Airborne
Surface
Settle_plate
Airborne
Corynebacteriales
Sphingobacteriia
Sphingobacteriales
Sphingobacteriaceae
Sphingobacterium
Gordoniaceae
Gordonia
Intrasporangiaceae
Janibacter
Janibacter_indicus
Gordonia_rubripertincta
Gordonia_sp_135
Gordonia_terrae
Janibacter_sp_YB324
Janibacter_limosus
Gordonia_sp_KTR9
Gordonia_phthalatica
Propionibacteriaceae
Gordonia_sp_HY186
Rhodococcus_pyridinivorans
Rhodococcus_sp_2G
Janibacter_melonis
Tessaracoccus
Mycolicibacterium_litorale
Rhodococcus_rhodochrous
Mycolicibacterium_phocaicum
Brevibacterium_linens
Micropruina
Micropruina_glycogenica
Tessaracoccus_flavescens
Micrococcales
Micrococcaceae
Kocuria
Kocuria_sp_RT304
Kocuria_rhizophila
Sporichthyaceae
Epidermidibacterium_keratini
Epidermidibacterium
Sporichthyales
Kocuria_rosea
Kocuria_palustris
Arthrobacter
Brevibacteriaceae
Brevibacterium
Kocuria_flava
Micrococcus
Micrococcus_luteus
Bacillus
Bacillus_cereus
0
1
2
3
4
5
LDA SCORE (log 10)

B

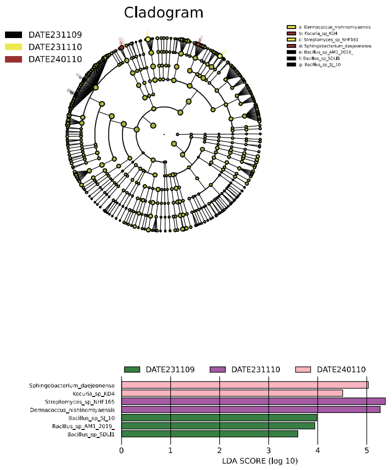
Cladogram
DATE231109
DATE231110
DATE240110
a: Dermacoccus_nishinomiyaensis
b: Kocuria_sp_KD4
c: Streptomyces_sp_NHF165
d: Sphingobacterium_daejeonense
e: Bacillus_sp_AM1_2019_
f: Bacillus_sp_SDLI1
g: Bacillus_sp_SL_10
DATE231109
DATE231110
DATE240110
Sphingobacterium_daejeonense
Kocuria_sp_KD4
Streptomyces_sp_NHF165
Dermacoccus_nishinomiyaensis
Bacillus_sp_SL_10
Bacillus_sp_AM1_2019_
Bacillus_sp_SDLI1
0
1
2
3
4
5
LDA SCORE (log 10)

C

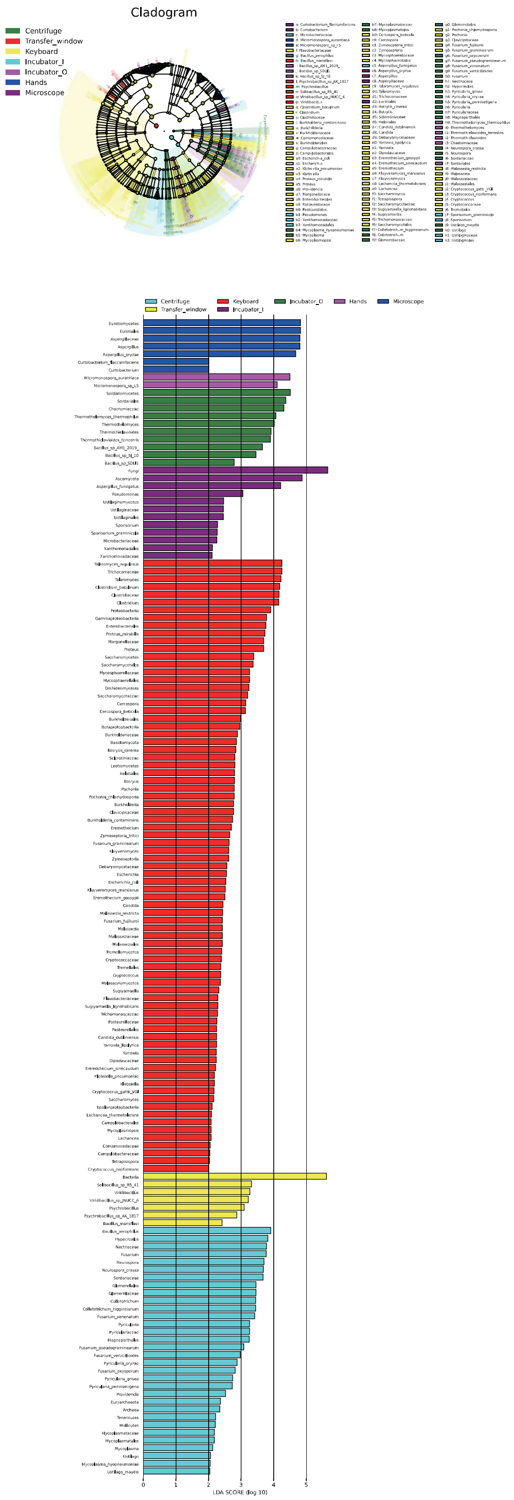
Cladogram
Centrifuge
Transfer_window
Keyboard
Incubator_I
Incubator_O
Hands
Microscope
Centrifuge
Transfer_window
Keyboard
Incubator_I
Incubator_O
Hands
Microscope
0
1
2
3
4
5
LDA SCORE (log 10)

Supplementary Figure 2. LEfSe analysis of species. (A) Grouped by sampling method, (B) by date, (C) by surface type. Upper panel: Linear discriminant analysis Effect Size analysis to identify species that best explain differences between two or more groups (referred to as biological markers or features), and the impact of these species on the differences. LEfSe is suitable for multi-level discovery and explanation, such as taxonomic phylogeny. The top 300 species by abundance are selected for drawing. Lower panel: LEfSe LDA value distribution bar chart. The y-axis shows the differentiated taxonomic units, the x-axis shows the LDA values, with higher LDA values indicating a greater contribution to the differences between groups. The colors of the bars represent the groups. The chart only displays LDA values above a set threshold (threshold of 2). Positive and negative values on the x-axis only indicate direction.

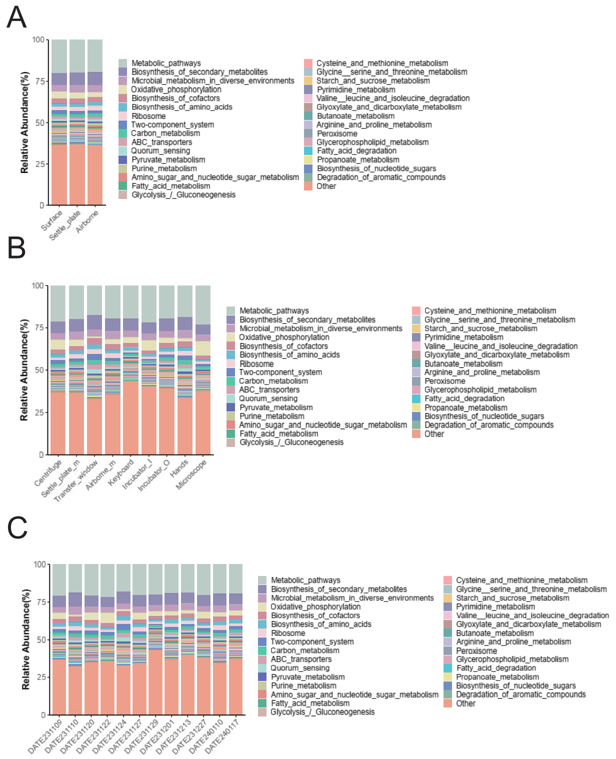
A
Relative Abundance(%)
100
75
50
25
0
Surface
Settle_plate
Airborne
Metabolic_pathways
Biosynthesis_of_secondary_metabolites
Microbial_metabolism_in_diverse_environments
Oxidative_phosphorylation
Biosynthesis_of_cofactors
Biosynthesis_of_amino_acids
Ribosome
Two-component_system
Carbon_metabolism
ABC_transporters
Quorum_sensing
Pyruvate_metabolism
Purine_metabolism
Amino_sugar_and_nucleotide_sugar_metabolism
Fatty_acid_metabolism
Glycolysis_/_Gluconeogenesis
Cysteine_and_methionine_metabolism
Glycine__serine_and_threonine_metabolism
Starch_and_sucrose_metabolism
Pyrimidine_metabolism
Valine__leucine_and_isoleucine_degradation
Glyoxylate_and_dicarboxylate_metabolism
Butanoate_metabolism
Arginine_and_proline_metabolism
Peroxisome
Glycerophospholipid_metabolism
Fatty_acid_degradation
Propanoate_metabolism
Biosynthesis_of_nucleotide_sugars
Degradation_of_aromatic_compounds
Other
B
Relative Abundance(%)
100
75
50
25
0
Centrifuge
Settle_plate_m
Transfer_window
Airborne_m
Keyboard
Incubator_I
Incubator_O
Hands
Microscope
C
Relative Abundance(%)
100
75
50
25
0
DATE231109
DATE231110
DATE231120
DATE231122
DATE231124
DATE231127
DATE231129
DATE231201
DATE231213
DATE231227
DATE240110
DATE240117

Supplementary Figure 3. Stacked bar charts of function abundance at level 3 of the Kegg database. (A) Grouped by sampling method, (B) by sampling method and surface type subgroup, (C) by date. The stacked bar charts show the x-axis as the sample/group and the y-axis as the relative abundance of functions, with the colors of the bars representing function classifications. The longer the bar, the higher the relative abundance.

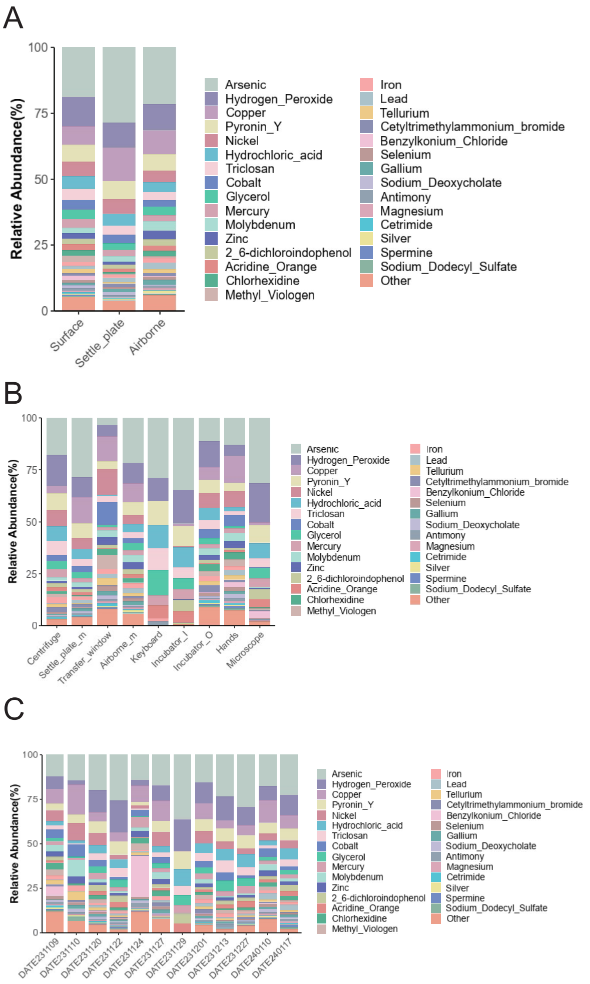
A
Relative Abundance(%)
100
75
50
25
0
Surface
Settle_plate
Airborne
Arsenic
Hydrogen_Peroxide
Copper
Pyronin_Y
Nickel
Hydrochloric_acid
Triclosan
Cobalt
Glycerol
Mercury
Molybdenum
Zinc
2_6-dichloroindophenol
Acridine_Orange
Chlorhexidine
Methyl_Viologen
Iron
Lead
Tellurium
Cetyltrimethylammonium_bromide
Benzylkonium_Chloride
Selenium
Gallium
Sodium_Deoxycholate
Antimony
Magnesium
Cetrimide
Silver
Spermine
Sodium_Dodecyl_Sulfate
Other
B
Relative Abundance(%)
Centrifuge
Settle_plate_m
Transfer_window
Airborne_m
Keyboard
Incubator_I
Incubator_O
Hands
Microscope
C
Relative Abundance(%)
DATE231109
DATE231110
DATE231120
DATE231122
DATE231124
DATE231127
DATE231129
DATE231201
DATE231213
DATE231227
DATE240110
DATE240117

Supplementary Figure 4. Stacked bar charts of function abundance from the BacMet database. (A) Grouped by sampling method, (B) by sampling method and surface type subgroup, (C) by date. The stacked bar charts show the x-axis as the sample/group and the y-axis as the relative abundance of functions, with the colors of the bars representing function classifications. The longer the bar, the higher the relative abundance.

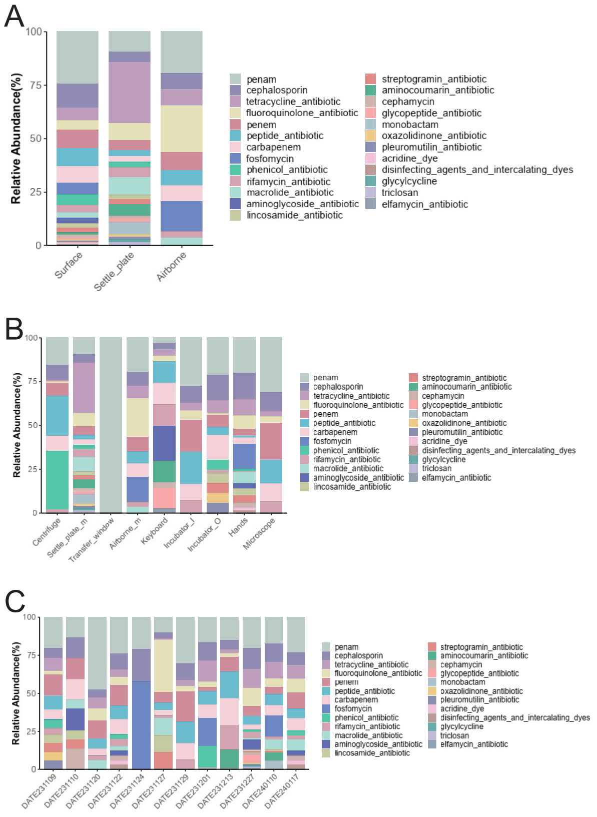

A
Relative Abundance(%)
100
75
50
25
0
Surface
Settle_plate
Airborne
penam
cephalosporin
tetracycline_antibiotic
fluoroquinolone_antibiotic
penem
peptide_antibiotic
carbapenem
fosfomycin
phenicol_antibiotic
rifamycin_antibiotic
macrolide_antibiotic
aminoglycoside_antibiotic
lincosamide_antibiotic
streptogramin_antibiotic
aminocoumarin_antibiotic
cephamycin
glycopeptide_antibiotic
monobactam
oxazolidinone_antibiotic
pleuromutilin_antibiotic
acridine_dye
disinfecting_agents_and_intercalating_dyes
glycylcycline
triclosan
elfamycin_antibiotic
B
Relative Abundance(%)
Centrifuge
Settle_plate_m
Transfer_window
Airborne_m
Keyboard
Incubator_I
Incubator_O
Hands
Microscope
C
Relative Abundance(%)
DATE231109
DATE231110
DATE231120
DATE231122
DATE231124
DATE231127
DATE231129
DATE231201
DATE231213
DATE231227
DATE240110
DATE240117

Supplementary Figure 5. Stacked bar charts of function abundance from the Card database. (A) Grouped by sampling method, (B) by sampling method and surface type subgroup, (C) by date. The stacked bar charts show the x-axis as the sample/group and the y-axis as the relative abundance of functions, with the colors of the bars representing function classifications. The longer the bar, the higher the relative abundance.

1 Surface_Grade A_Inside Biosafety Cabinet (Cab-I)

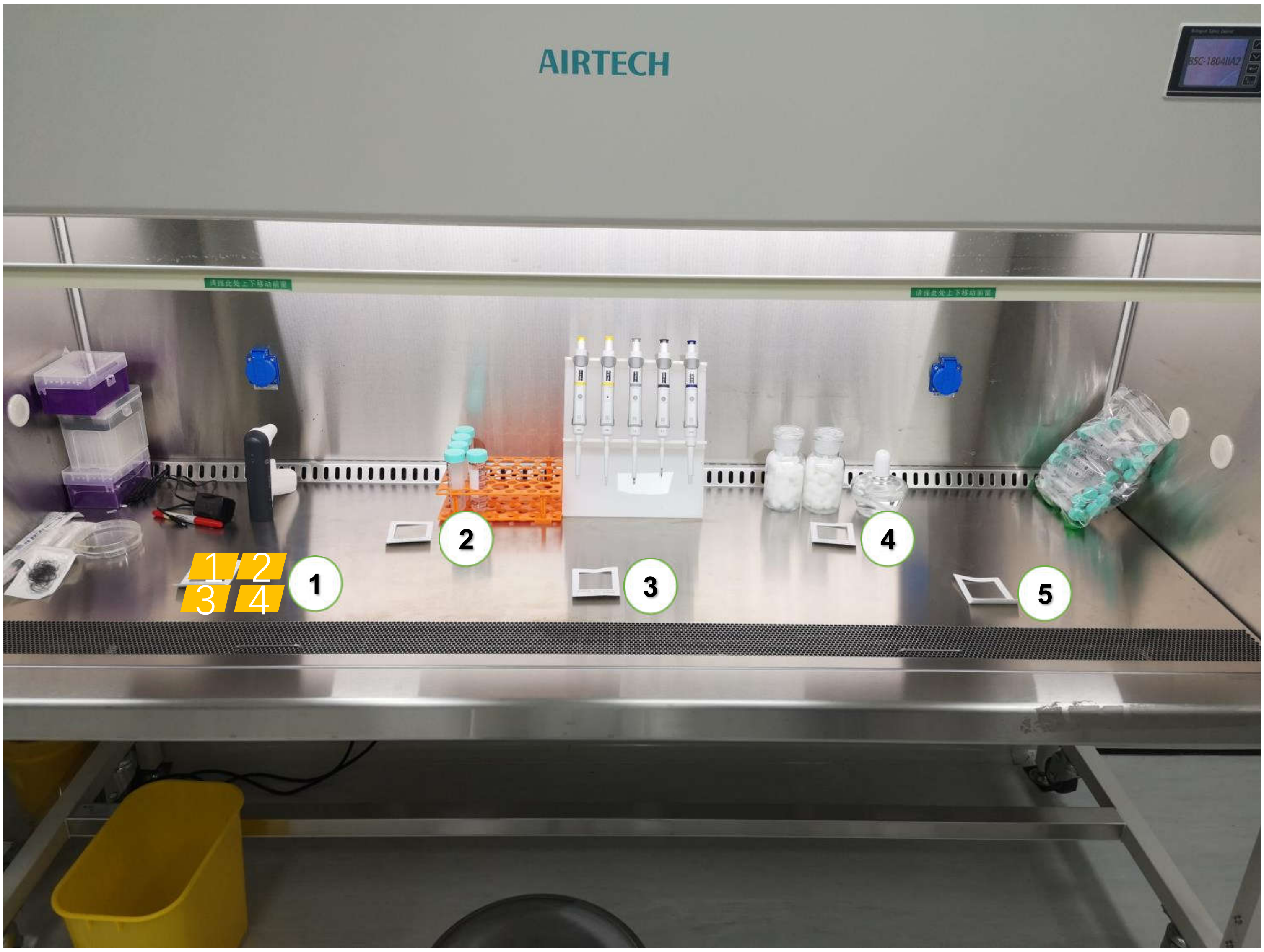

Supplementary Figure 6. Surface Sampling Diagram - Inside Biosafety Cabinet (Grade A Environment): Five sampling points are designated, with each point sampled four times using a sterile 5 cm × 5 cm template. Related to Figure 1A.

1 Surface_Grade B_Outside Biosafety Cabinet (Cab-O)

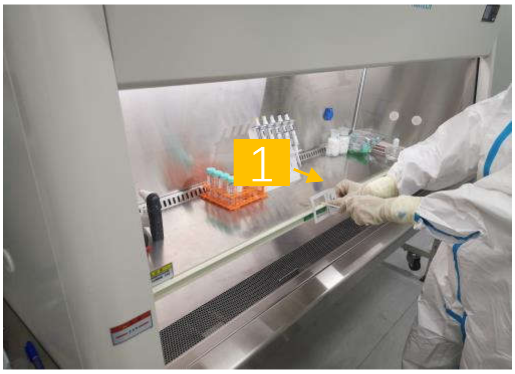

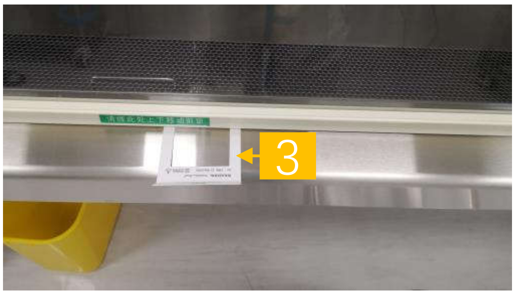

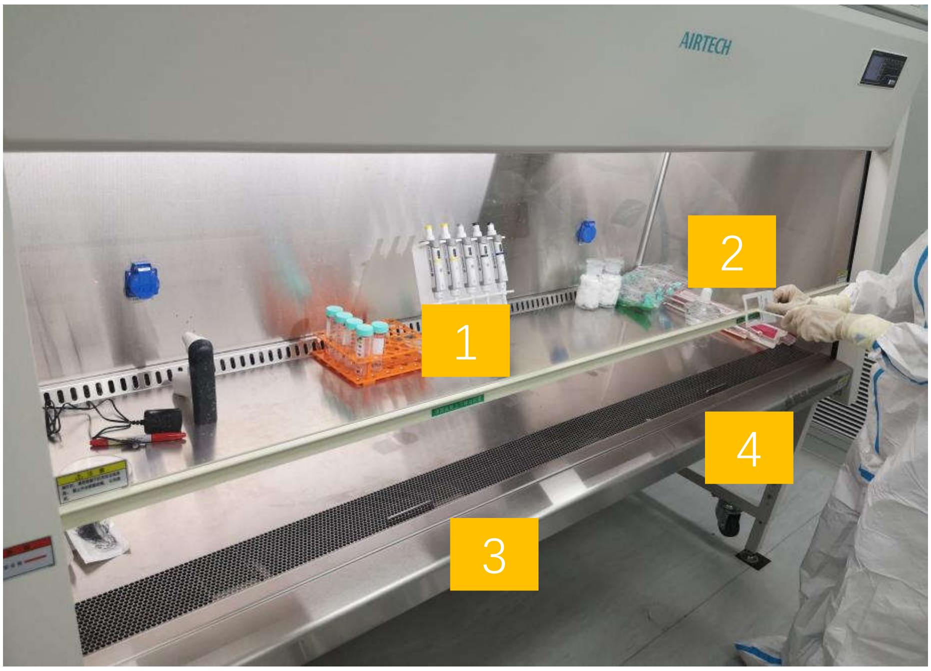

Supplementary Figure 7. Surface Sampling Diagram - Outside Biosafety Cabinet (Grade B Environment): One sampling point is designated, with the point sampled four times using a sterile 5 cm × 5 cm template, as indicated by the yellow arrows or circles in the figure. Related to Figure 1B.

2 Surface_Grade B_Outside Incubator

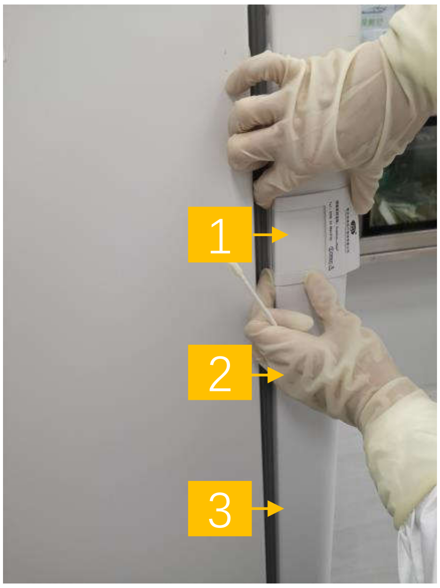

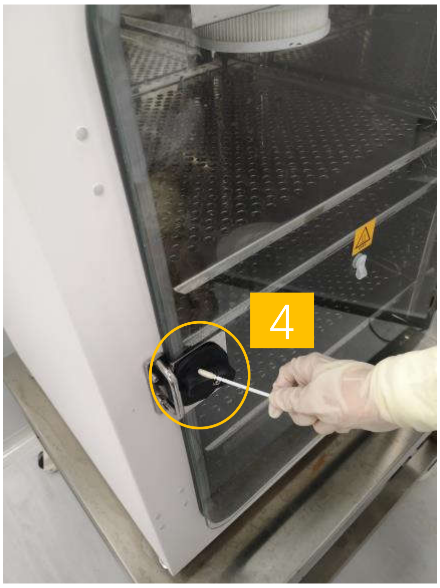

Supplementary Figure 8. Surface Sampling Diagram - Outside Incubator (Grade B Environment): One sampling point is designated, with the point sampled four times using a sterile 5 cm × 5 cm template, as indicated by the yellow arrows or circles in the figure. Related to Figure 1B.

3 Surface_Grade B_Inside Incubator (Cab-I)

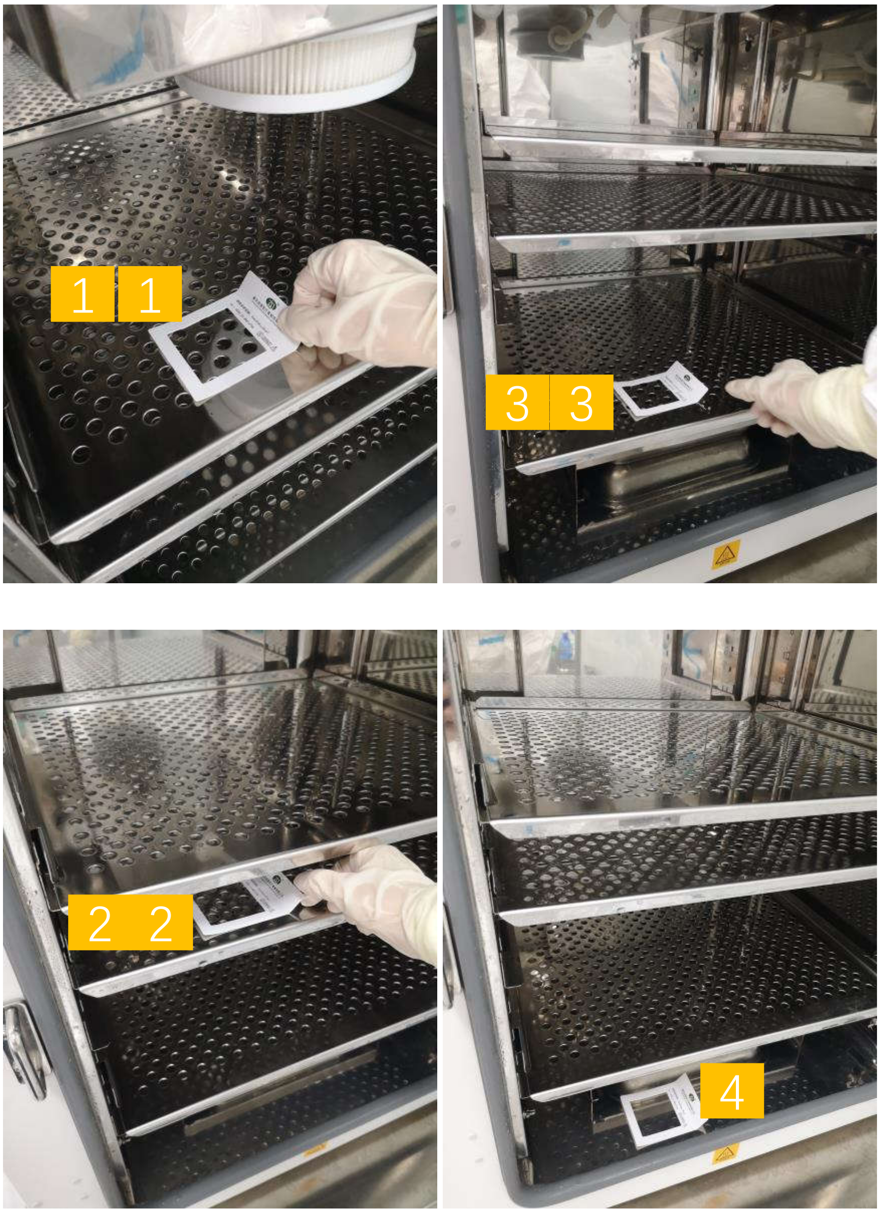

Supplementary Figure 9. Surface Sampling Diagram - Inside Incubator (Grade B Environment): One sampling point is designated. This point is sampled four times using a template. The first three samples each cover two template areas (due to the perforated surface), and the fourth sample covers one template area. Related to Figure 1B.

4 Surface_Grade B_Transfer Window

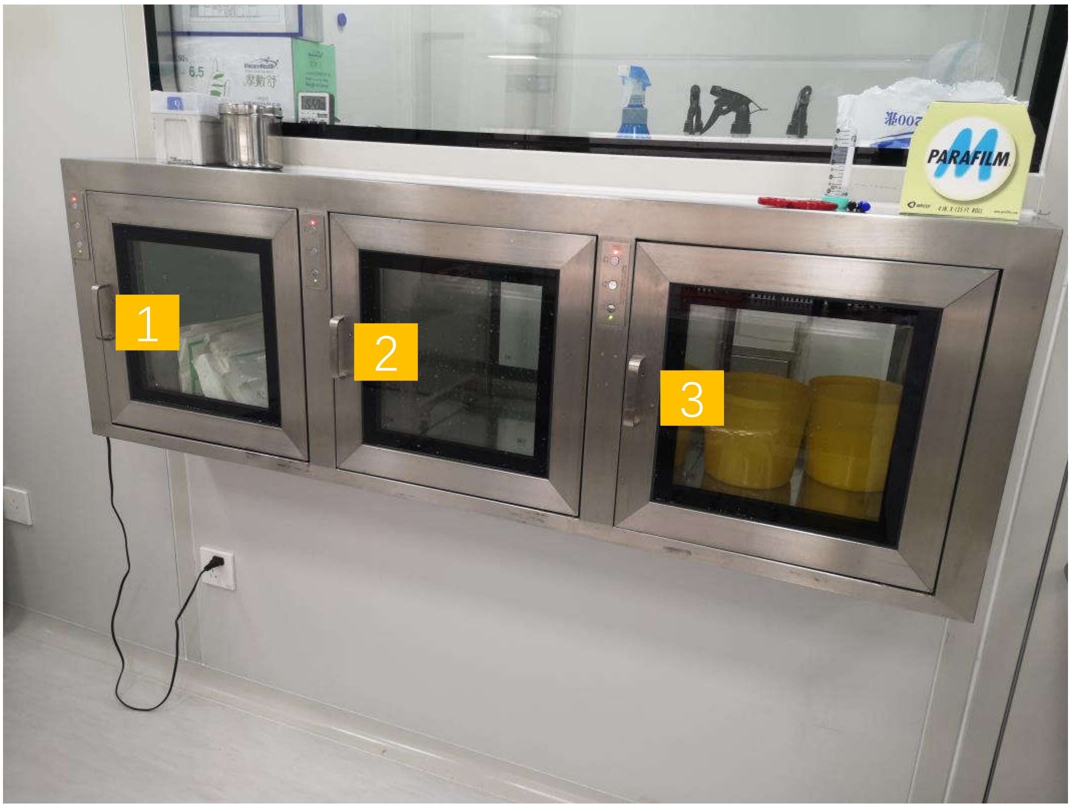

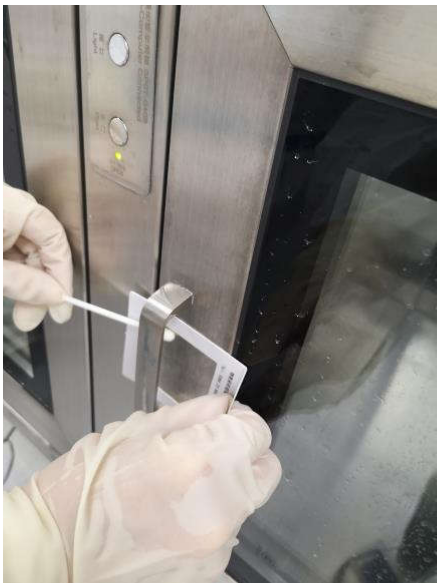

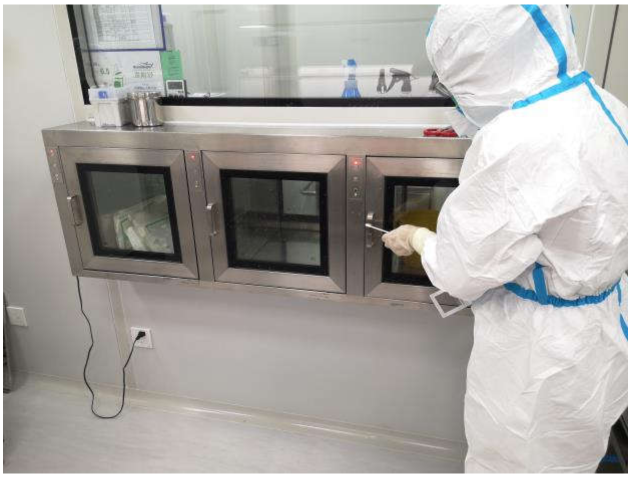

Supplementary Figure 10. Surface Sampling Diagram - Transfer Window (Grade B Environment): One sampling point is designated. This point is sampled using a template for the cabinet surface and without a template for the handles. Each of the three handles is sampled once, and the corresponding positions on the cabinet surface are sampled once each using a template. Related to Figure 1B.

5 Surface_Grade B_Keyboard (mouse included)

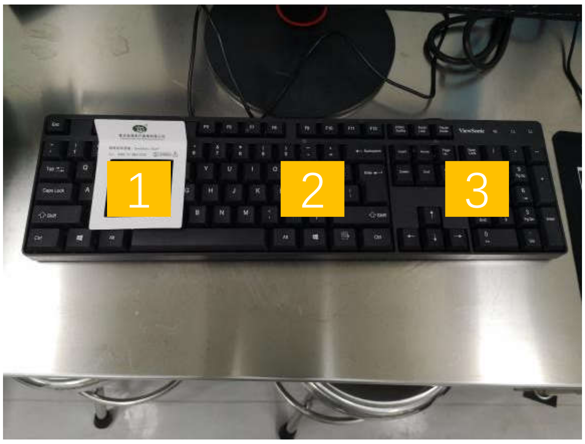

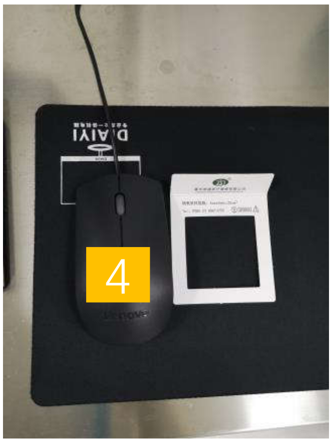

Supplementary Figure 11. Surface Sampling Diagram - Outside Incubator (Grade B Environment): One sampling point is designated, with the point sampled four times using a sterile 5 cm × 5 cm template, as indicated by the yellow square in the figure. Related to Figure 1B.

6 Surface_Grade B_Microscope

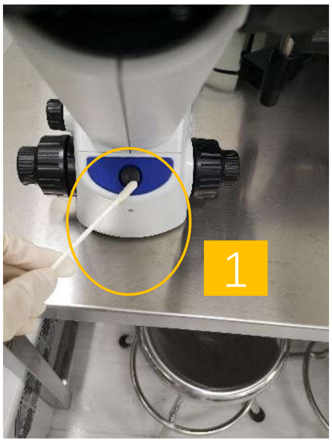

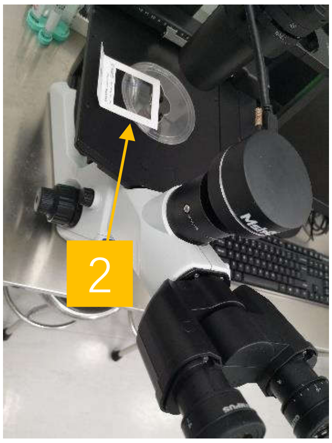

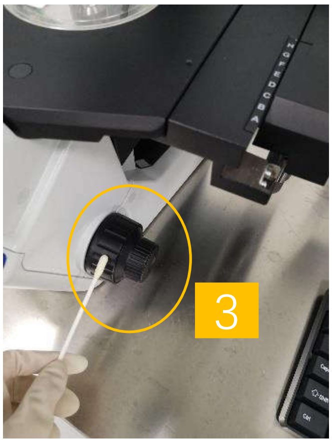

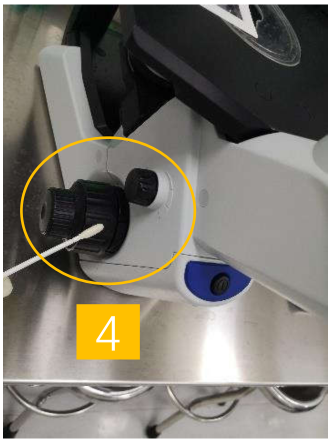

Supplementary Figure 12. Surface Sampling Diagram - Microscope (Grade B Environment): One sampling point is designated, with the point sampled four times using a sterile 5 cm × 5 cm template, as indicated by the yellow arrows or circles in the figure. Related to Figure 1B.

7 Surface_Grade B_Centrifuge

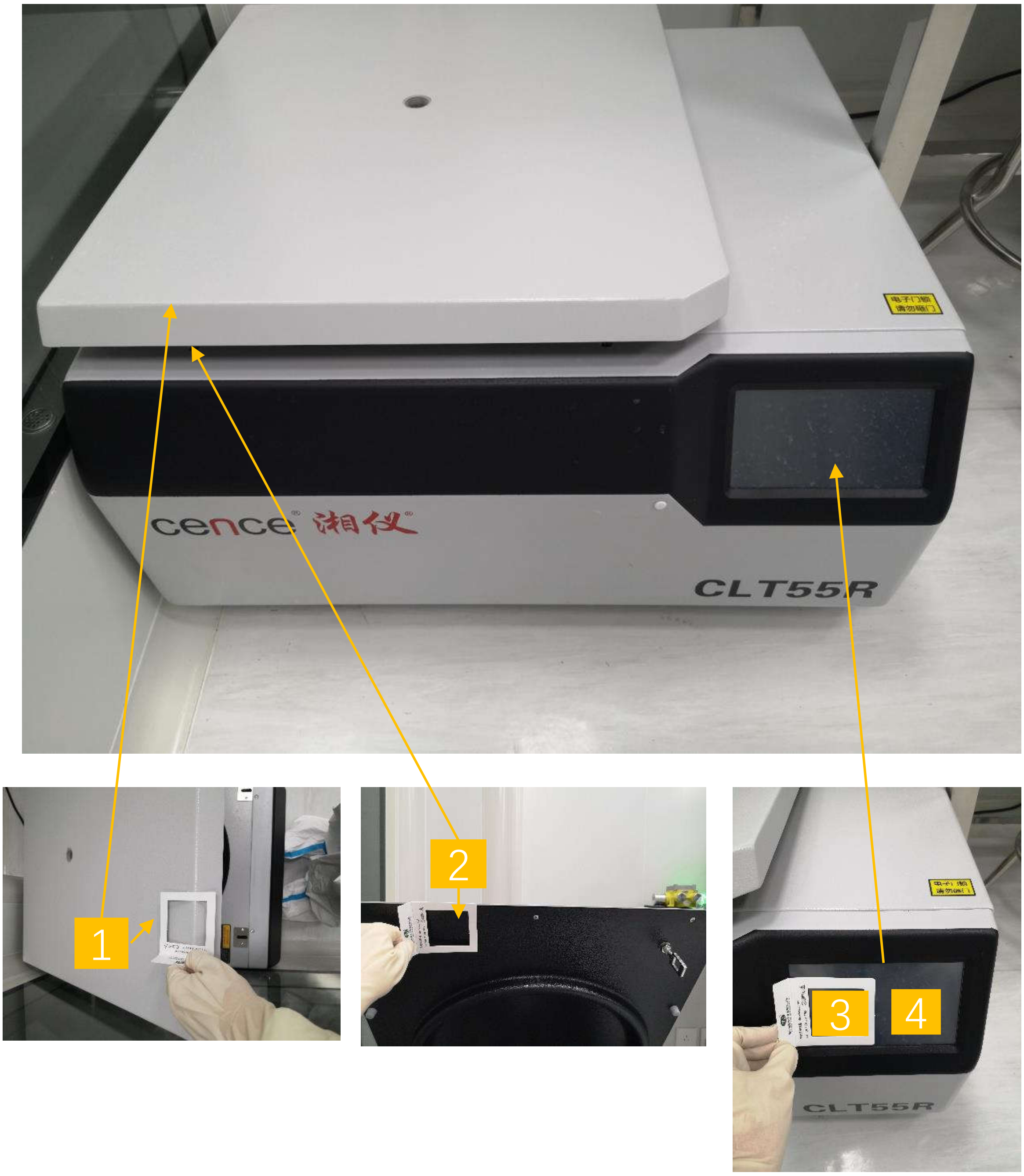

Supplementary Figure 13. Surface Sampling Diagram - Centrifuge (Grade B Environment): One sampling point is designated, with the point sampled four times using a sterile 5 cm × 5 cm template, as indicated by the yellow arrows in the figure. Related to Figure 1B.
